# Targeting of Interferon-Beta to Produce a Specific, Multi-Mechanistic Oncolytic Vaccinia Virus

**DOI:** 10.1371/journal.pmed.0040353

**Published:** 2007-12-27

**Authors:** David H Kirn, Yaohe Wang, Fabrice Le Boeuf, John Bell, Steve H Thorne

**Affiliations:** 1 Jennerex Biotherapeutics, San Francisco, California, United States of America; 2 Clinical Pharmacology, University of Oxford, Oxford, United Kingdom; 3 Cancer Research UK Molecular Oncology Centre, Queen Mary's School of Medicine and Dentistry, Charterhouse Square, London, United Kingdom; 4 Ottawa Health Research Institute, Ottawa, Ontario, Canada; 5 Department of Pediatrics and Bio-X Program, Stanford University, Stanford, California, United States of America; University of Florida, United States of America

## Abstract

**Background:**

Oncolytic viruses hold much promise for clinical treatment of many cancers, but a lack of systemic delivery and insufficient tumor cell killing have limited their usefulness. We have previously demonstrated that vaccinia virus strains are capable of systemic delivery to tumors in mouse models, but infection of normal tissues remains an issue. We hypothesized that interferon-beta (IFN-β) expression from an oncolytic vaccinia strain incapable of responding to this cytokine would have dual benefits as a cancer therapeutic: increased anticancer effects and enhanced virus inactivation in normal tissues. We report the construction and preclinical testing of this virus.

**Methods and Findings:**

In vitro screening of viral strains by cytotoxicity and replication assay was coupled to cellular characterization by phospho-flow cytometry in order to select a novel oncolytic vaccinia virus. This virus was then examined in vivo in mouse models by non-invasive imaging techniques. A vaccinia *B18R* deletion mutant was selected as the backbone for IFN-β expression, because the *B18R* gene product neutralizes secreted type-I IFNs. The oncolytic *B18R* deletion mutant demonstrated IFN-dependent cancer selectivity and efficacy in vitro, and tumor targeting and efficacy in mouse models in vivo. Both tumor cells and tumor-associated vascular endothelial cells were targeted. Complete tumor responses in preclinical models were accompanied by immune-mediated protection against tumor rechallenge. Cancer selectivity was also demonstrated in primary human tumor explant tissues and adjacent normal tissues. The *IFN-β* gene was then cloned into the *thymidine kinase* (*TK*) region of this virus to create *JX-795* (*TK^−^/B18R^−^/IFN-β^+^*). *JX-795* had superior tumor selectivity and systemic intravenous efficacy when compared with the TK^−^/B18R^−^ control or wild-type vaccinia in preclinical models.

**Conclusions:**

By combining IFN-dependent cancer selectivity with IFN-β expression to optimize both anticancer effects and normal tissue antiviral effects, we were able to achieve, to our knowledge for the first time, tumor-specific replication, IFN-β gene expression, and efficacy following systemic delivery in preclinical models.

## Abstract 2

Stephen Thorne and colleagues describe, in a mouse model, an oncolytic vaccinia virus with interferon-dependent cancer selectivity that allows tumor-specific replication; it also expresses the IFN-β gene and hence has efficacy against tumors.

## Editors' Summary

**Background.:**

Normally, throughout life, cell division (which produces new cells) and cell death are carefully balanced to keep the body in good working order. But sometimes cells acquire changes (mutations) in their genetic material that allow them to divide uncontrollably to form cancers—disorganized masses of cells. Cancers can develop anywhere in the body and, as they develop, their cells acquire other genetic changes that enable them to move and start new tumors (metastases) elsewhere. Chemotherapy drugs kill rapidly dividing cancer cells but, because some normal cells are also sensitive to these drugs, it is hard to destroy the cancer without causing serious side effects. Consequently, researchers are trying to develop “targeted” therapies that attack the changes in cancer cells that allow them to divide uncontrollably but leave normal cells unscathed. One promising class of targeted therapies is oncolytic viruses. These viruses make numerous copies of themselves inside cancer cells (but not inside normal cells). Eventually the cancer cell bursts open (lyses), releases more of the therapeutic agent, and dies.

**Why Was This Study Done?:**

Existing oncolytic viruses have two major disadvantages: they have to be injected directly into tumors, and therefore they can't destroy distant metastases; and they don't kill cancer cells particularly efficiently. In this study, the researchers have tried to adapt vaccinia virus (a virus that infects humans and which has recently been shown to kill tumor cells when injected into the bloodstream) in two ways: to both infect cancer cells selectively and then to kill them effectively.

They hypothesized that putting a gene that causes expression of a protein called interferon-beta (IFN-β) in a particular virus strain that is itself incapable of responding to IFN-β might achieve these aims. Human cells infected with viruses usually release IFNs, which induce an antiviral state in nearby cells. But vaccinia virus makes anti-IFN proteins that prevent IFN release. If the viral genes that encode these proteins are removed from the virus, the virus cannot spread through normal cells. However, many cancer cells have defective IFN signaling pathways so the virus can spread through them. IFN-β expression by the virus, however, should improve its innate anticancer effects because IFN-β stops cancer cells dividing, induces an antitumor immune response, and stops tumors developing good blood supplies.

**What Did the Researchers Do and Find?:**

The researchers selected a vaccinia virus strain called WR-delB18R in which the B18R gene, which encodes an anti-IFN protein, had been removed from the virus. (WR is a wild-type virus.) In laboratory experiments, IFN treatment blocked the spread of WR-delB18R in normal human cells but not in human tumor cells. After being injected into the veins of tumor-bearing mice, WR-delB18R was rapidly cleared from normal tissues but persisted in the tumors. A single injection of WR-delB18R directly into the tumor killed most of the tumor cells. A similar dose injected into a vein was less effective but nevertheless increased the survival time of some of the mice by directly killing the tumor cells, by targeting the blood supply of the tumors, and by inducing antitumor immunity. Finally, when the researchers inserted the *IFN-β* gene into this WR-delB18R, the new virus—JX-795—was much better at killing tumors after intravenous injection than either WR or WR-delB18R.

**What Do These Findings Mean?:**

These findings indicate that the vaccinia virus can be adapted so that it replicates only in tumor cells and kills these cells effectively after intravenous injection. In particular, they show that the strategy adopted by the researchers both optimizes the anticancer effects of the virus and minimizes viral replication in normal tissues. JX-795 is a promising oncolytic virus, therefore, particularly since vaccinia virus has been safely used for many years to vaccinate people against smallpox. Nevertheless, it will be some years before JX-795 can be used clinically. Vaccinia virus constructs like this need to be tested extensively in the laboratory and in animals before any attempt is made to test them in people and, even if they passes all these preclinical tests with flying colors, only clinical trials will reveal whether they can treat human cancer. Several related strains of vaccinia virus are currently undergoing clinical testing.

**Additional Information.:**

Please access these Web sites via the online version of this summary at http://dx.doi.org/:10.1371/journal.pmed.0040353.

- The US National Cancer Institute provides information on all aspects of cancer (in English and Spanish)
- CancerQuest, from Emory University, provides information on all aspects of cancer (in several languages)
- The UK charity Cancerbackup also provides information on all aspects of cancer
- Wikipedia has a page on oncolytic viruses (note that Wikipedia is a free online encyclopedia that anyone can edit; available in several languages)
- A short interview about oncolytic viruses with researcher Dr. John Bell is available on the Insidermedicine Web site
- The Oncolytic virus Web page provides lists of oncolytic viruses classified by type

## Introduction

Oncolytic viruses have promise as cancer therapeutics due to their targeted nature and ability to destroy cancer cells through novel mechanisms-of-action (oncolysis and/or necrosis) [1–3]. Selective intratumoral replication of the virus leads to viral multiplication and spread to adjacent cancer cells and subsequent lysis of all infected cancer cells. One targeting strategy that has proven successful in a variety of oncolytic strains involves deletions of viral anti–type-I interferon (IFN) gene products. Cancer selectivity results through IFN-mediated inhibition of replication in normal tissues, whereas replication and oncolysis proceeds unhindered in tumor cells with defects in IFN responses [4–7]. Examples include mutations of the M-protein genes in vesicular stomatitis virus (VSV), the gamma-34.5 genes in herpes simplex virus (HSV), and viral-associated (VA) RNA in adenovirus. In addition, a variety of unmodified small RNA viruses have demonstrated natural tumor tropism mediated by their inherent sensitivity to the interferon-mediated antiviral state in normal cells [4,8,9].

Oncolytic viruses can further be engineered to express therapeutic transgene products that can destroy tumors through diverse and complementary mechanisms [10,11]. One such transgene, *IFN-β*, has multiple anticancer effects, including direct antiproliferative effects [12], the induction of tumor-specific cytotoxic T lymphocytes (CTL) [13], and antiangiogenic effects [14]. IFN-β protein therapy is approved for use in recurrent multiple sclerosis [15] and phase I–II clinical trials have been performed with IFN-β in patients with brain tumors and other metastatic solid tumors [16]. However, overall efficacy was limited and transient, and significant systemic toxicities limited further dose escalation [17].

We hypothesized that expression of IFN-β from an oncolytic virus would have dual benefits by increasing anticancer efficacy and increasing safety. Although IFN-β in normal tissues inhibits viral replication [18], tumor cells are commonly resistant to the antiviral effects of type-I IFNs; oncolytic virus replication should therefore not be inhibited in these IFN-resistant cancer cells. Nevertheless, IFN-β–mediated anticancer effects, including the induction of tumor-specific CTL and antiangiogenic effects should still be operative. In order to achieve safe, sustained, high-level expression of IFN-β selectively in tumor tissue, we engineered IFN-β expression from a tumor-selective oncolytic vaccinia virus. Expression of IFN-β required a novel approach, however, because vaccinia expresses multiple gene products that block type-I IFN responses [19,20]. Of particular importance, vaccinia expresses a secreted inhibitor of type-I IFN designated B18R [21,22]. We hypothesized that deletion of *B18R* would be necessary to allow expressed IFN-β to function effectively, and that deletion of this gene could also lead to enhanced tumor selectivity.

## Methods

### Viruses and Cell Lines

Vaccinia strain Western Reserve (WR) was purchased from ATCC. The *B18R*-deleted strain of WR was kindly provided by Professor Geoff Smith (Imperial College, London, United Kingdom). *B18R*, *thymidine kinase* (*TK*) double-deleted viruses were constructed by insertion of DNA into the vaccinia *TK* gene by homologous recombination. The cloning plasmid pSC65 (provided by Professor Bernie Moss, National Institutes of Health) was remade so that the firefly *luciferase* gene was expressed from the pSE/L promoter and, for *JX-795*, the murine beta-interferon (mIFN-β) cDNA was cloned for expression from the p7.5 early/late promoter. The mIFN-β cDNA was provided by Mike Parr (Biogen-Idec). Successful recombination events were selected for by *luciferase* expression, and correct insertion of plaque-purified clones was verified by PCR. Recombination (producing *TK* inactivation) was performed into both *B18R*-deleted virus or WR, using vectors expressing luciferase alone, or luciferase and mIFN-β. Myxoma virus (strain Lausanne) (MV) was kindly provided by Professor Grant McFadden (University of Florida).

Primary human cells (small airway epithelial cells [SAECs] and normal human bronchial epithelial cells [NHBEs]) were purchased from Clonetics (Lonza Biosciences); C33A, A2780, and HCT 116 human tumor cell lines, BSC-1 (monkey kidney cells), and NIH 3T3 murine cell lines were purchased from ATCC and CMT-93; JC and CMT-64 (murine tumor cell line) were provided by Cancer Research UK cell bank collection.

### In Vitro Replication, Viability, Bioluminescence, and ELISA Assays

Cells were treated with human IFN-α (50 U/ml; SIGMA) in six-well plates either 24 h prior to or 5 h postinfection with different viruses. Viruses were added at a multiplicity of infection (MOI) of 1.0 viral plaque forming unit (PFU)/cell. Virus (from cells and medium) was collected 72 h later (unless otherwise indicated) and titered after three rounds of freezing and thawing on BSC-1 cells by plaque assay.

In cell viability assays, serial dilutions of virus were added to cells in 96-well plates, and IFN-α was added 5 h later. Cell viability was determined 72 h later by MTS assay (Promega), and viral MOI required to reduce cell viability to 50% effective concentration (EC50) (relative to untreated controls [100%] or cell-free wells [0%]) was determined from a standard curve.

Bioluminescence assays were performed following infection of cell layers in six-well plates at an MOI of 1.0 or 0.05 with viral strains expressing firefly luciferase. After 24h, luciferin substrate (30 mg/ml; Caliper) was added to the wells and bioluminescence determined in an IVIS50 system (Caliper).

ELISA for murine IFN-β was performed following infection of cell layers in six-well plates at an MOI of 1.0 PFU/ml with viral strains expressing mIFN-β. Every hour after infection, the medium was changed, and the spent medium spun (300*g*, 5 min) to remove dead cells, and retained to quantify mIFN-β produced within the previous hour. ELISA for mIFN-β (Bioscource) was performed according to the manufacturer's instructions.

ELISA for human IFN-β was performed following treatment of cell layers in six-well plates with lipopolysaccharide (LPS; 5μg/ml), or infection with viruses (modified vaccinia virus Ankara [MVA] or *WR-delB18R*) at an MOI of 1.0 PFU/ml. Medium was collected 18 h after treatment, filtered (0.22 μm) to remove cells or virus, and ELISA (Fujirebio) run according to the manufacturer's instructions.

### Flow Cytometry

Cells were grown in six-well plates, before human IFN-β was added (100 U/ml; SIGMA). Fifteen minutes later, cells were fixed by addition of paraformaldehyde (to 1.6%), scraped, washed and permeabilized in 100% methanol, before a second wash. Cells were stained with rabbit anti-phospho-STAT1 (Tyr701; Cell Signaling Technology), with an APC-labeled secondary, and samples run on a FACScaliber (Becton Dickinson).

### Explant Preparation, Culture, and Infection

Fresh tumor and adjacent normal tissue specimens were sliced into approximately 2-mm^3^ pieces and placed on a Surgifoam sponge that was presoaked with alpha medium containing 10% fetal bovine serum. *B18R* and *TK* gene–deleted vaccinia virus encoding green fluorescent protein (GFP) was then added directly to the specimen (1 × 10^7^ PFU) and allowed to infect for 2 h at 37 °C before covering the sponge and specimen with medium containing serum. At 48 h, specimens were visualized using fluorescence microscopy.

### In Vivo Biodistribution, Bioluminescence, and Efficacy Assays

Tumors were formed by subcutaneous implantation of syngeneic tumor cells into immunocompetent mice. A total of 5 × 10^5^ CMT-93 or JC tumor cells were implanted into C57/B6 or BALB/c mice respectively. Tumors were allowed to form for 10–14 d (until they reached 50–100 mm^3^ as determined by caliper measurements), animals were then regrouped and treated with a single intravenous (tail vein) or intratumoral injection of 1 × 10^8^ PFU of virus (unless otherwise stated). During the biodistribution studies, animals were sacrificed at indicated times after treatment; organs were recovered and snap frozen. Organs were then homogenized and viral titers determined by plaque assay on BSC-1 cells.

In some experiments, animals were treated with virus expressing firefly luciferase. Animals were imaged using an IVIS100 system (Caliper) 5 min after intraperitoneal injection of 150 mg/kg luciferin (Caliper). Animals were anesthetized with 2% isoflurane. Regions of interest were drawn around the whole animal and the tumor, and light output determined using the LivingImage software (Caliper). Light output for the torso was determined as the whole-body signal minus the tumor signal.

In the efficacy experiments, tumor burden was determined by caliper measurement at indicated times after treatment, and animals sacrificed once tumors reached 1,500 mm^3^. Survival curves (Kaplan-Meier) were plotted.

All animal studies were performed under a UK project license, or with US Institutional Animal Care and Use Committee (IACUC) approval.

### Ultrasound Analysis of Tumor Vasculature

Mice bearing subcutaneous JC tumors implanted onto the flank were imaged using the Vevo770 small-animal ultrasound system (VisualSonics) during the delivery of a 100-μl bolus of Vevo MicroMarker nontargeted contrast agent (VisualSonics) through the tail vein. Reference data collected prior to delivery of the contrast agent was used to determine areas of blood flow within the tumor.

### Ex Vivo Immunohistochemistry, Immunofluorescence, and ELISA

Some animals bearing subcutaneous tumors and treated with different viruses (as above) were sacrificed and their tumors fixed in paraformaldehyde, embedded in paraffin, sectioned (5 μm), and then stained for immunohistochemistry. A polyclonal anti-vaccinia or a monoclonal anti-mouse CD3 antibody was used to detect viral-infected cells or CD3-positive cells, and visualized using horseradish peroxidase (DAKO). As a control, vaccinia strain WR was UV-inactivated following treatment with psoralen, and then column purified.

In similar experiments, tumors treated with vaccinia strains expressing GFP were frozen in optimal temperature control (OTC) compound, sectioned (10 μm), fixed in acetone, and stained with anti-CD31 antibody conjugated to phycoerythrin (PE; BD Pharmigen). GFP and PE fluorescence was examined on a Leica Confocal microscope.

ELISA was also run to detect mIFN-β on serum collected by retino-orbital bleeding, or from tumor tissue. Tumor tissue was weighed, homogenized, and then cleared by gentle centrifugation before ELISA for mIFN-β (Biosource).

### Statistical Analyses

Kaplan-Meier curves were compared using the log rank test. Analyses of all other data were performed using a Student *t*-test.

## Results

### In Vitro Tumor Selectivity of a *B18R* Gene-Deleted Vaccinia (*WR-delB18R*)

**Figure 1:**
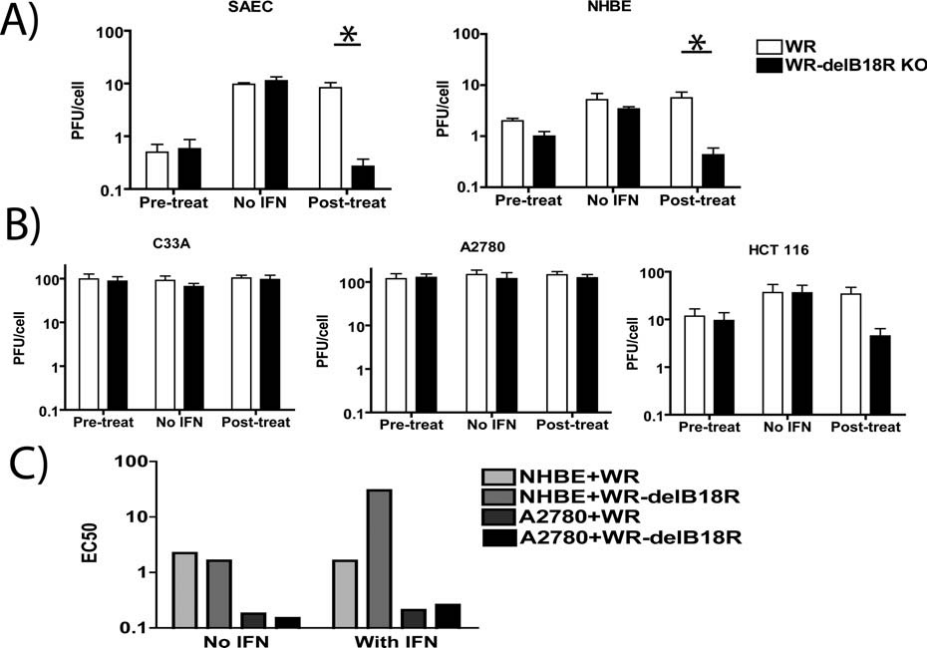
Effect of Type-I IFN on Replication of Vaccinia Strains in Tumor and Normal Cell Lines (A) Primary human cell lines (SAECs and NHBEs) were grown to 50% confluence in six-well plates and treated with human IFN-α (50 U/ml) either 24 h prior to or 5 h after infection with vaccinia (or else PBS was used as a control). Vaccinia strains WR (white bars) or *WR-delB18R* (WR with deletion of the *B18R* gene; black bars) were used at an MOI of 1.0 PFU/cell. After 72 h, viral titers in the wells were determined by plaque assay (Student *t*-test for WR versus *WR-delB18R* with IFN treatment postinfection, *p* = 0.0055 for SAECs and 0.0012 for NHBEs). (B) This assay was repeated using human tumor cell lines C33A, A2780, and HCT 116. (C) Serial dilutions of vaccinia strains were added to cells (NHBE or A2780) 5 h before addition of human IFN-alpha (or PBS) as before. Cell viability in the different wells was determined 72 h later by MTS assay, and EC50 values (viral PFU/ml required to reduce cell viability to 50% of untreated well) were determined. An asterisk (*) indicates significant difference (*p* = 0.0055 for SAECs and 0.0012 for NHBEs).

Because a variety of viral strains unable to, or deleted in their ability to, interfere with the type-I IFN response are also tumor targeting, we hypothesized that vaccinia strains containing deletions in genes acting on this pathway would also display tumor-specific replication. We therefore screened WR and a strain of WR vaccinia virus containing a deletion in the *B18R* gene (*WR-delB18R*) against several human cancer cell lines and primary cells. The *B18R* gene product is a secreted or membrane-bound type-I IFN-binding protein that is known to block the protective effects of type-I IFN on vaccinia-infected cells and whose deletion increases the median lethal dose (LD50) of the virus by more than three logs [22]. It is therefore capable of binding and inactivating type-I IFN that has been secreted from infected and/or adjacent cells, and thereby prevents both the induction of an antiviral state in surrounding cells, and induction of an antiviral immune response. It was found that addition of type-I IFN (human IFN-α) to the primary cells prior to infection induced an antiviral state in these cells and limited viral replication in a one-step replication study (Figure 1A). However, addition of the IFN after viral infection was only effective in producing an antiviral state in the *B18R*-deleted strain, which was unable to inhibit the extracellular cytokine (*p* = 0.0055 for SAECs and 0.0012 for NHBEs). When the tumor cell lines C33A and A2780 were similarly treated, no effect of IFN addition was seen under any conditions, implying an inability of these cells to respond to this cytokine (Figure 1B). In HCT 116 cells, however, it was found that addition of IFN-β postinfection was capable of reducing replication of the *B18R*-deleted strain (*WR-delB18R*) relative to WR.

It was similarly demonstrated that IFN addition postinfection with *WR-delB18R* could also protect primary cells, but not IFN-resistant tumor cells, from viral-mediated cell killing (Figure 1C) and that this effect was dependent on loss of *B18R*. It was therefore possible to increase the tumor selectivity and therapeutic index of vaccinia in vitro through *B18R* deletion.

**Figure 2:**
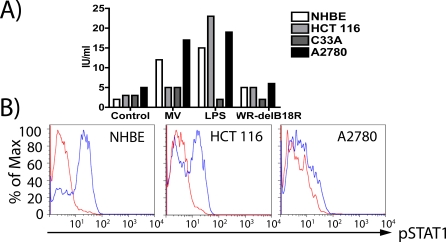
Dysfunction in Type-I IFN Production or Signaling in Normal and Tumor Cell Lines (A) Production of IFN-β. Cell lines (NHBEs, HCT 116, C33A, and A2780) were infected with MV or *WR-delB18R* at an MOI of 5.0, or treated with LPS at 5 μg/ml; medium was collected after 18 h, and IFN-β levels determined by ELISA. (B) Response to IFN-β. Cell lines (NHBEs, HCT 116, and A2780) were treated with IFN-β (blue graphs) or PBS (red graphs), and fixed and permeabilized 15 min later. Levels of phospho-STAT1 were determined by flow cytometry.

The precise nature of any dysfunction in IFN signaling in the A2780 and C33A cells and their response to viral exposure were also investigated and compared to the NHBE and HCT 116 cell lines. Although HCT 116 has previously been reported to be capable of responding to type-I IFN [23], this response was not in the context of an antiviral response. The ability of these cell lines to produce, or respond to, IFN-β was therefore examined (Figure 2). IFN-β production in the presence of a viral pathogen may be in response to stimuli from within an infected cell (typically through PKR induction by dsRNA for DNA viruses), or external stimuli (typically detected through binding to Toll Like Receptors (TLR)). Primary cells (NHBEs) were found to up-regulate IFN-β secretion in response to either MV infection (which has previously been demonstrated to produce IFN-β upon infection of human fibroblasts, probably through PKR activation [24]) or following exposure to LPS (which, along with viral glycolipids, binds to and activates TLR4) (Figure 2A). However, NHBEs did not produce IFN-β above background levels when infected with *WR-delB18R* (Figure 2A). This lack of IFN-β production is not surprising because vaccinia expresses several genes that interfere with PKR activation and signaling (e.g., *E3L* and *K3L*). It was further found that A2780 cells could also increase IFN-β production in response to either MV infection or LPS exposure (but not to *WR-delB18R* infection); that HCT 116 could respond to LPS exposure, but could not respond to MV infection; and that C33A was unable to increase IFN-β production under any of the conditions examined.

The ability of these cells to respond to IFN-β addition was also examined through phosphorylation of STAT1, an integral step in the signaling pathway following binding of type-I IFNs to their receptor (Figure 2B). NHBEs were found to be able to respond to IFN-β exposure, as were HCT 116 cells; however, A2780 cells, although able to produce IFN-β, were unable to respond to it. It therefore appears that (1) all vaccinia-infected cells are blocked in their ability to produce type-I IFN directly (even though both NHBEs and A2780 can produce type-I IFN in response to infection with the related MV); (2) C33A is unable to produce IFN in response to TLR activation; and (3) A2780 cells are unable to respond directly to type-I IFN. The ability of different tumor and normal cells to produce or respond to type-I IFN is therefore closely associated to their ability to replicate the *B18R*-deleted virus.

### Selectivity and Efficacy of the *B18R* Deletion Mutant In Vivo

**Figure 3:**
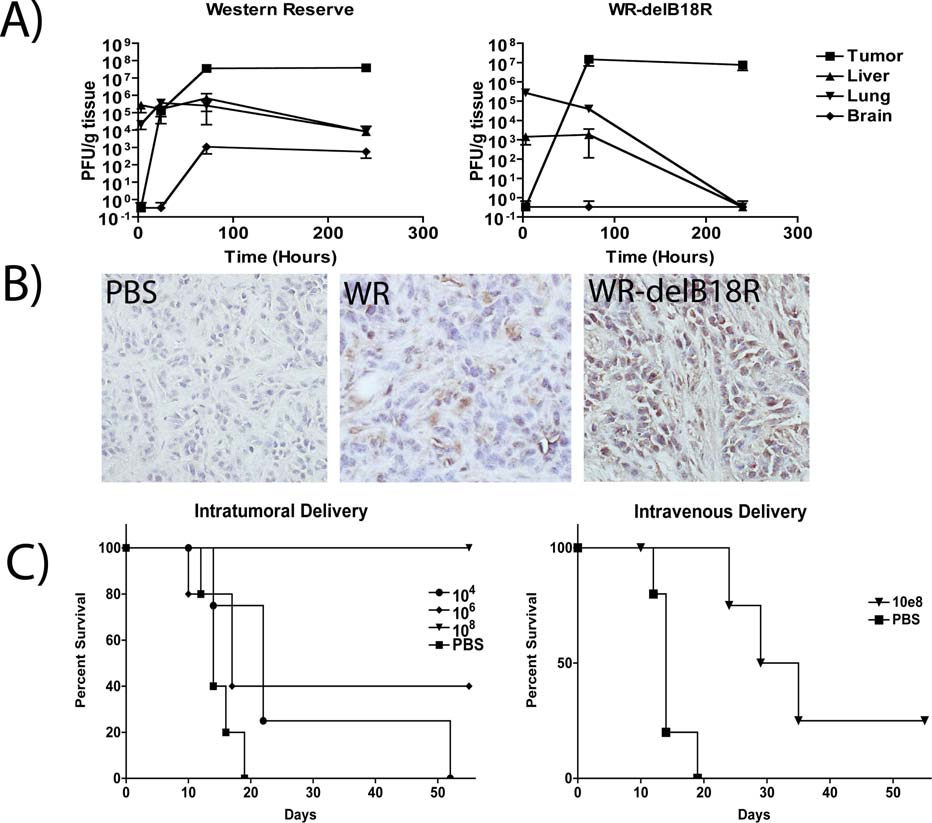
Systemic Delivery, Selectivity, and Oncolytic Activity of *WR-delB18R* Virus (A) Immunocompetent (BALB/c) mice bearing JC tumors were treated via a single tail vein injection with 1 × 10^8^ PFU of vaccinia strains WR or *WR-delB18R*. Viral titer (PFU/g) in indicated tissues was determined by plaque assay after sacrifice of animals at indicated time points (*n* = 3 animals/time point). (B) Immunohistochemistry staining for vaccinia coat proteins in tumor tissue sections of animals treated as above and sacrificed 24 h after viral treatment (magnification 40×). (C) Survival of immunocompetent mice (C57/B6) bearing subcutaneous CMT-93 tumors and treated when tumors reached 50–100 mm^3^ with a single intratumoral (left) or intravenous (right) injection of *WR-delB18R* or PBS. Doses of 1 × 10^4^ (circles), 1 × 10^6^ (diamonds), or 1 × 10^8^ (triangles) PFU of virus or PBS controls (squares) were used (*n* = 5 mice/group; *p* = 0.0047 for intravenous injections).

Immunocompetent mice bearing syngeneic subcutaneous tumors were treated with sublethal doses (1 × 10^8^ PFU) of wild-type WR or *WR-delB18R* by a single intravenous injection (the *B18R* gene product is known to bind both murine and human type-I IFNs [22]). Animals were sacrificed at times post-treatment, and infectious virus recovered from different organs was titered (Figure 3A). The *B18R*-deleted strain was rapidly cleared from all tissues other than the tumor, where it persisted at levels equivalent to the wild-type WR virus for the duration of the study (Figure 3B). Of particular note, no *B18R*-deleted virus was recovered from the brain at any time point.

Efficacy of *WR-delB18R* as a single agent was also tested following intratumoral or intravenous delivery (Figure 3C). A dose-dependent response to intratumoral injection was seen, with a single injection of 1 × 10^8^ PFU of virus, resulting in 100% complete responses. This same dose given intravenously was also capable of producing significant improvements in survival over control animals (*p* = 0.0047) and some complete responses, but in a smaller number of the treated mice. Higher doses, though feasible, were not tested.

### Multi-Mechanistic Tumor Killing by *WR-delB18R* Vaccinia Virus

**Figure 4:**
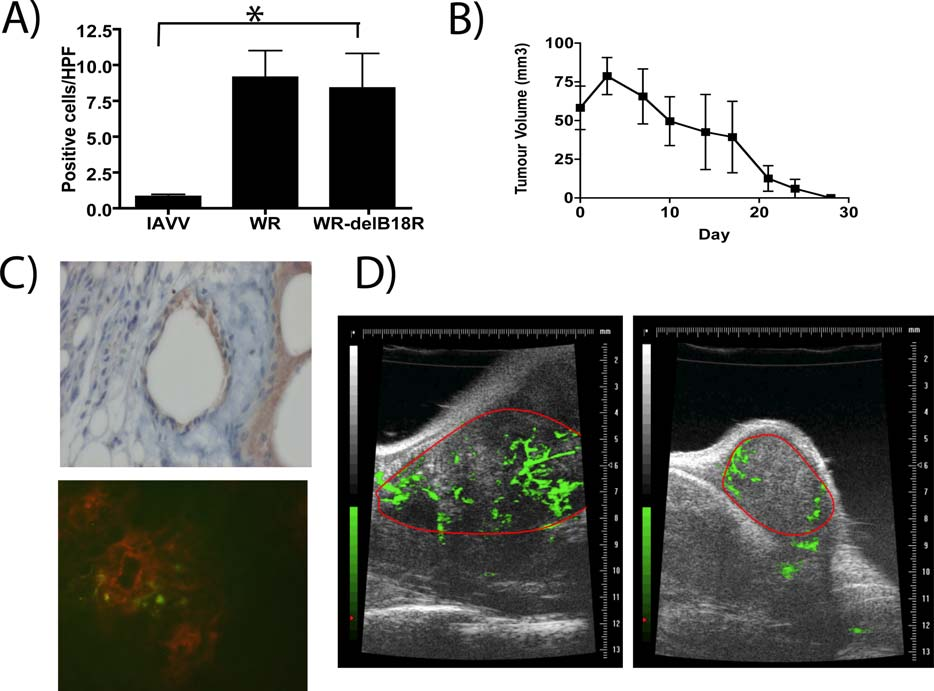
Potential Mechanisms of Tumor Cell Killing Employed by *WR-delB18R* (A) Immunocompetent (C57/B6) mice bearing subcutaneous CMT-93 tumors and treated with a single tail vein injection of 1 × 10^8^ PFU of either psoralen-UV–inactivated WR (IAVV), WR, or *WR-delB18R* were sacrificed 7 d post-treatment, and CD4-positive cells in tumor sections were scored blind by a pathologist. Average numbers of positive cells per high-powered field (40×) from an average of ten randomly chosen fields from each of three mice treated under each condition are represented (*p* = 0.035 for IAVV compared to *WR-delB18R* treated). An asterisk (*) indicates significantly different counts. (B) Mice treated as in Figure 3C and displaying complete responses were rechallenged with a subcutaneous injection of 5 × 10^5^ CMT-93 cells. Tumor burden was measured by calipers (*n* = 8 mice). (C) Mice (C57/B6 bearing subcutaneous CMT-93 tumors) were treated with a single tail vein injection of 1 × 10^8^ PFU of *WR-delB18R* virus and sacrificed after 24 h. Tumor sections stained for viral coat proteins indicated initial infection of tumor vascular endothelial cells (top), whereas tumors from mice treated as above, but with *WR-delB18R* expressing GFP (WR-ΔB18RΔTK-GFP), were examined by immunofluorescence following staining with PE-conjugated antibody targeting CD31 (endothelial cells) (bottom; green = viral GFP expression; red = endothelial cell CD31 staining). (D) Vascular collapse in tumors of mice treated with *WR-delB18R*. Subcutaneous CMT-93 tumors implanted into C57/B6 mice were examined by ultrasound immediately prior to (left) and 48 h after (right) intravenous treatment with 1 × 10^8^ PFU *WR-delB18R*. A nonspecific contrast agent was delivered intravenously during ultrasound data acquisition in order to detect tumor vasculature (green); the border of the tumor was manually delineated (red).

It was hypothesized that immune-mediated destruction of infected tumor cells may help enhance the oncolytic effect of the virus. As such, it was noted that a significant increase in the numbers of tumor-infiltrating lymphocytes was seen in all animals treated with replication-competent viruses (*p* = 0.035) (Figure 4A). In addition, animals that had undergone complete responses in Figure 3C, when rechallenged with the same tumor cell line, were able to reject the tumor implant, implying that infected tumor cells may be capable of cross-presenting tumor antigens and thereby elicit a protective antitumor immune response (Figure 4B).

Finally, we noted that intravenously delivered virus infected not only tumor cells initially, but also tumor vascular endothelial cells (Figure 4C), resulting in viral gene expression in and around the endothelial cells. It is likely that infection would lead to destruction of the infected tumor endothelial cells, with resultant intravascular thrombosis and vascular collapse within the tumor mass. The infection and subsequent destruction of tumor endothelial cells was seen as a loss of vascular density in a treated animal within 48 h of intravenous delivery of *WR-delB18R* (Figure 4D). This tumor-associated vascular targeting may represent a previously undescribed mechanism of tumor destruction mediated by the *B18R*-deleted strain and, presumably, other vaccinia strains. Of note, this finding was not observed in any of the normal organ vasculatures assessed histologically.

### Selectivity of the *B18R* Deletion in Human Tumor Explant Tissue

**Figure 5:**
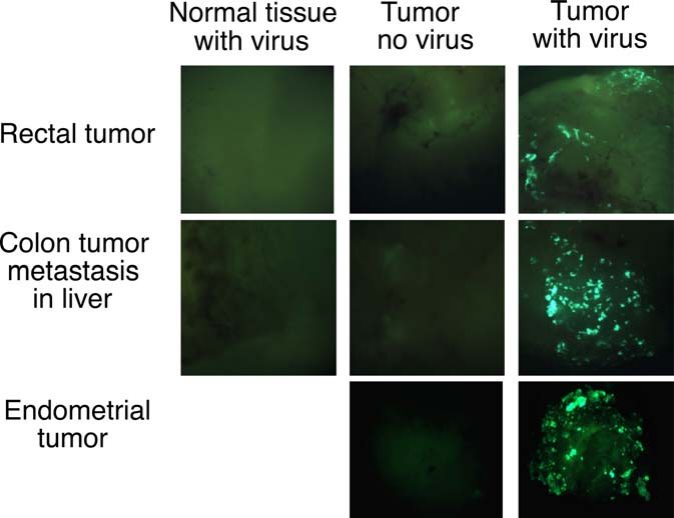
Infection of Primary Tumor and Normal Explant Tissues with *B18R-* and *TK*- Deleted Virus Expressing GFP Three tissue samples (top: rectal tumor with normal rectal tissue; middle: colon tumor metastases in liver; and bottom: endometrial tumor [no normal tissue available]:) are represented. Tumor and normal tissue were mixed with virus, washed, and images taken 48 h later.

In order to establish whether the tumor-targeting potential of the *B18R* deletion was also relevant in primary tumor tissue, infection of explant tissues was examined. For this work, the *GFP* gene was inserted into the viral *TK* gene of *WR-delB18R* in order to track viral infection and gene expression. The *TK* gene was chosen both because standard cloning strategies exist to target it, and because the *TK* gene deletion is itself tumor targeting [25]. It was clearly seen that this virus was capable of successfully infecting several tumor types, but was incapable of gene expression from either normal liver or colorectal tissue (Figure 5). Whereas only background level of virus (1 × 10^3^ PFU/g) was recovered from the normal liver tissue after 48 h, 1 × 10^8^ PFU/g was recovered from the colon tumor metastasis in the adjacent tissue (unpublished data), indicating the *GFP* gene expression translates to successful viral replication.

### Characterization of the IFN-β–Expressing *TK* ^−^/*B18R* ^−^ Vaccinia Mutant (*JX-795*) In Vitro

Because the vaccinia *B18R* gene product normally binds and removes secreted type-I IFNs, including IFN-β, a *B18R* deletion mutant would be the optimal vaccinia strain to express this cytokine. We constructed a cassette containing the murine *IFN-β* gene and the firefly *luciferase* gene under the control of the vaccinia p7.5 and pSE/L promoters, respectively. This cassette was inserted by homologous recombination into the viral *TK* gene of *WR-delB18R*.

**Figure 6:**
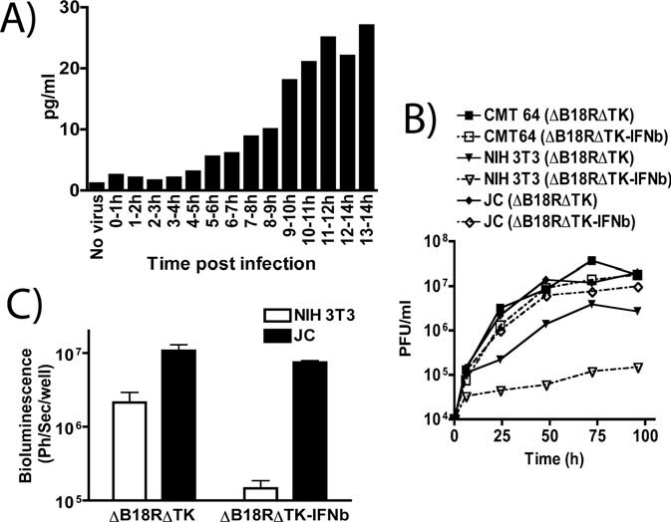
In Vitro Testing of *JX-795* (Western Reserve Vaccinia Virus Containing Deletions of *B18R* and *Thymidine Kinase* Genes and Expressing Luciferase and mIFN-β) (A) mIFN-β production following infection of human A2780 cells with *JX-795* at an MOI of 5.0. IFN-β secretion into the medium during each hour postinfection (up to 14 h) was determined by ELISA. (B) Replication of *JX-795* (solid symbols and lines; ΔB18RΔTK-IFNb) and an equivalent virus (*B18R*- and *TK*-deleted, expressing luciferase only; Δ18ΔTK: open symbols; dashed lines) following infection of murine tumor (CMT-64 [squares] and JC [diamonds]) or nontransformed (NIH 3T3; triangles) cells at an MOI of 1.0. (C) Viral gene expression (luciferase) as measured by bioluminescence 24 h after infection of indicated cell lines with indicated viruses at an MOI of 1.0, and quantified (average of three experiments).

Significant levels of IFN-β expression appeared within 5 h after infection of a tumor cell monolayer, and reached a plateau by 11 h postinfection; the plateau concentration was approximately 10-fold higher than at 5 h (Figure 6A). These data are consistent with primarily late transgene expression, as would be expected with the vaccinia p7.5 early/late promoter driving gene expression [26]. This implies that high levels of gene expression are linked to replication of the viral genome, and therefore will not occur efficiently during infection of resistant normal cell types. This adds an extra layer of safety, as expression from viral early/late promoters allows for low levels of gene expression early in infection, even during infection of cells that do not support viral replication. This allows normal tissues to mount a type-I IFN–dependent antiviral response early after exposure to virus, thus preventing high-level late gene expression leading to IFN-mediated toxicity.

Therefore, because *JX-795* produces its own IFN-β, additional tumor selectivity should be achieved without addition of the cytokine exogenously. This was shown to be the case in several murine cell lines (Figure 6B); both *JX-795* and an equivalent, IFN-β–negative control virus (carrying deletions in both *B18R* and the viral *TK* gene, but not expressing IFN-β; *TK* ^−^/*B18R* ^−^) could replicate in tumor cell lines to equivalent levels. However, when the nontransformed NIH 3T3 cell line was used, viral expression of IFN-β reduced viral replication by several logs. This also demonstrates a dysfunction in the type-I IFN response for the murine CMT 64 and JC tumor cell lines. This finding was supported by assays of viral gene expression (Figure 6C). These assays indicated that, although the *B18R* and *TK* gene deletions resulted in approximately a 5-fold reduction in gene expression from nontransformed cells relative to tumor cells, the addition of IFN-β expression from this virus resulted in a two-log reduction in viral gene expression from the nonmalignant cells, without effecting the ability of the virus to replicate in tumor cells.

### *IFN-β* Gene Expression, Tumor Selectivity, and Efficacy of *JX-795* In Vivo

**Figure 7:**
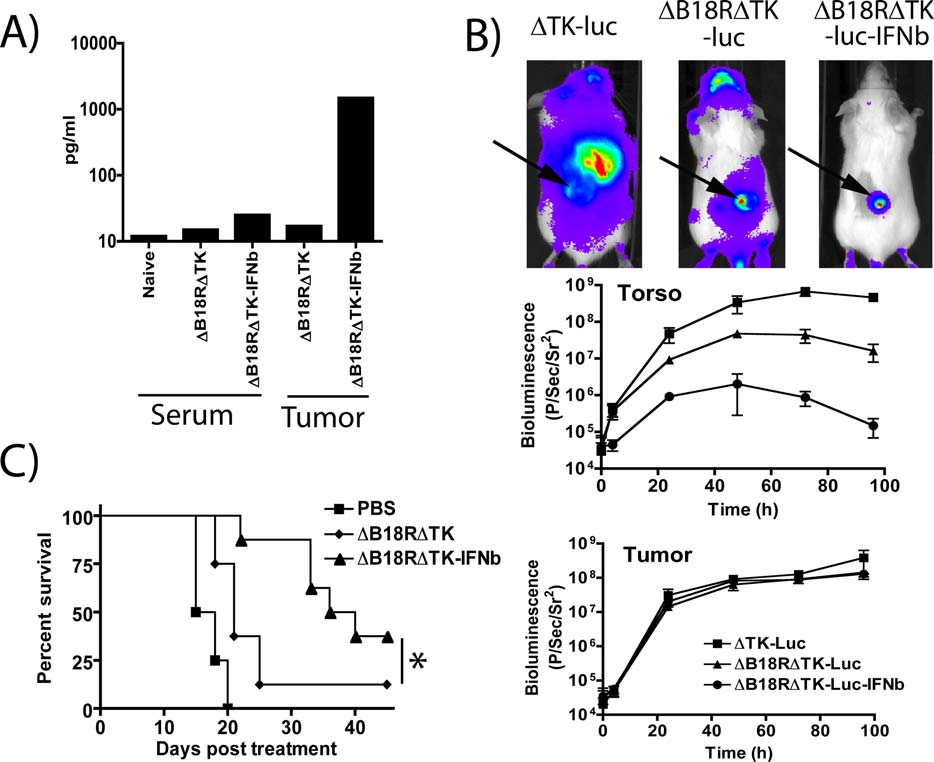
In Vivo Testing, Biodistribution, and Efficacy of *JX-795* (A) mIFN-β levels were determined by ELISA in the serum, and the tumors of animals (BALB/c bearing subcutaneous JC tumors) treated with (1 × 10^8^ PFU) *JX-795* (ΔB18RΔTK-IFNb), ΔB18RΔTK virus, or PBS 72 h earlier (*n* = 3/group). (B) Biodistribution of viral gene expression (as determined by bioluminescence imaging of mice treated with viruses expressing luciferase). Mice (BALB/c) bearing subcutaneous JC tumors (arrows) were treated with a single tail vein injection of 1 × 10^8^ PFU of viruses, *TK*-deleted (ΔTK-luc; squares), ΔB18RΔTK-luc (triangles), or ΔB18RΔTK-luc-IFNb (*JX-795*; circles), and imaging performed after luciferin addition. Representative images were taken at 72 h post-treatment. Bioluminescence was quantified over the tumor and the torso of the treated animals (*n* = 3/group) at times post-treatment (middle and bottom right-hand panels; symbols are for both graphs). (C) Survival curves of BALB/c mice bearing subcutaneous JC tumors and treated with a single tail vein injection of 1 × 10^8^ PFU of ΔB18RΔTK virus (diamonds), ΔB18RΔTK-IFNb (*JX-795*; triangles) or PBS (squares) (*p* = 0.028 for ΔB18RΔTK relative to ΔB18RΔTK-IFNb).

*JX-795* was delivered via tail vein injection to BALB/c mice bearing established (50–100 mm^3^) subcutaneous tumors. In initial experiments, the concentration of IFN-β in the serum and in tumors of the animals was assayed (Figure 7A). High levels of IFN-β were present in the tumors of the treated animals, but the cytokine appeared to remain localized; only very small increases in the serum levels of IFN-β were observed in these animals, which were not sufficient to produce myelosuppression or transaminitis in the animals (Table 1), or any overt signs of toxicity, such as signs of neurotoxicity. Furthermore, bioluminescence imaging (BLI) was used to determine the biodistribution of viral gene expression (Figure 7B). The *TK*-deleted virus infected primarily the tumor, but also to a lesser extent a variety of nontumor tissues within the limbs, abdomen, and head (nasal and brain) of the treated mice. In contrast, *TK* ^−^/*B18R* ^−^ double-deleted virus had a more restricted pattern of viral gene expression. *JX-795*, however, produced the greatest level of tumor selectivity with minimal if any gene expression appearing outside of the tumor. Quantification of this signal indicated that, whereas the *TK* ^−^/*B18R* ^−^ signal remained at least 10-fold lower than the *TK* ^−^ signal from 48 h postinfection onwards, the expression of IFN-β from this strain further reduced viral nontarget signal by almost two logs, and signal had returned to background levels by 96 h post-treatment. Direct correlation between in vivo bioluminescence from luciferase-expressing vaccinia and viral replication in different tissues has been demonstrated previously [27]. Quantification of viral gene expression from within the tumor target indicated that tumor infection levels were equivalent for all viruses, despite the marked attenuation of *JX-795* in nonmalignant tissues. *JX-795* may therefore be further used as a gene-delivery vector capable of selectively expressing further therapeutic transgenes from within the tumor following systemic delivery.

**Table 1:**
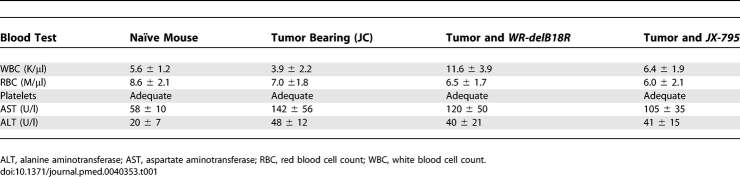
Blood Chemistry and Cell Counts for Mice Undergoing Different Treatments (*n* = 3/Group)

In order to determine the antitumor effects of the different viruses, immunocompetent BALB/c mice bearing JC tumors were treated with a single tail vein injection of 1 × 10^8^ PFU of different viruses or PBS control. *JX-795* produced significantly greater survival than any other group (*p* = 0.028), with three of eight animals displaying complete responses, demonstrating the additional antitumor benefits of expressing IFN-β (Figure 7C).

## Discussion

We report here that IFN-β expression from a *B18R*-deleted vaccinia results in a systemically effective, highly selective oncolytic virus. To our knowledge, this is the first time that such a rationally designed combination of attenuating viral gene deletion and transgene expression has been incorporated into a systemically deliverable vector, and the first successful report of systemic *IFN-β* gene delivery to a tumor.

Oncolytic viruses hold promise for the treatment of cancer, but improvements are needed [2]. In particular, systemic efficacy against metastatic tumors will be required in order to have a major impact on cancer patient survival. Novel approaches are therefore needed to improve intravenous safety and efficacy [28]. We hypothesized that IFN-β expression from an oncolytic vaccinia virus could achieve both objectives. First, intravenous delivery of vaccinia viruses appears to be feasible in immunocompetent animal tumor models [29,30]. Second, IFN-β antiviral effects in normal tissues should enhance safety. Finally, IFN-β expression in tumors should increase efficacy over that mediated by oncolysis alone.

Because type-I IFNs also possess antiviral properties, vaccinia has evolved to express both intracellular (K3L, E3L, and H1L) and extracellular (B18R) gene products that interfere with type-I IFN activity [20]. In particular, the viral *B18R* gene product is secreted from infected cells, binding and neutralizing extracellular IFN-α and -β [22]. Therefore, to prevent neutralization of IFN-β after secretion from infected cancer cells, we deleted the *B18R* gene from the vaccinia virus backbone used to express the *IFN-β* gene.

It appears that the *B18R*-deleted vaccinia virus (*WR-delB18R*) itself is capable of tumor-specific replication. Whereas all primary cells tested were capable of inducing an antiviral state when pretreated with type-I IFN, most (but not all) cancer cell lines were incapable of responding to this cytokine. However, when IFN-α was added 5 h postinfection, to better mimic the likely order of exposure in vivo, wild-type vaccinia (WR), but not *WR-delB18R*, was capable of preventing a subsequent block in viral replication in susceptible cells. As a result, *WR-delB18R* was attenuated in normal cells, but not in most tumor cells, when type-I IFN was added postinfection; this sequence would be expected to occur in vivo. Further examination of these effects revealed that in primary cells, *WR-delB18R*, like wild-type WR, was effective at preventing release of IFN-β from infected cells, but that uninfected neighboring cells could be induced to produce IFN-β (presumably through TLR binding), and so induce an antiviral IFN-β response. This antiviral response could be blocked by expression of *B18R* or by addition of anti–IFN-β neutralizing antibody (unpublished data), and was irrelevant in many cancer cells that were deficient in their ability to produce (C33A) or respond to (A2780 and C33A) IFN-β.

The oncolytic potential of *WR-delB18R* was confirmed in immunocompetent mice, with the virus rapidly removed from all tissues other than the tumor, and capable of producing 100% complete responses after local delivery. Antitumor effects were also seen following intravenous delivery, demonstrating the systemic potential of this virus. However, fewer complete responses were witnessed, indicating that an increase in tumor cell–killing potential for this virus (such as by transgene expression) may be needed for optimal systemic efficacy. Intravenous delivery also exposes more nonmalignant tissues and organs to the potential of viral infection, and so tumor-selectivity may become more critical for this delivery route. Although no toxicity was observed with *WR-delB18R* at therapeutic doses, deletion of intracellular IFN-resistance genes may lead to similar effects as *B18R* deletion, and the combination of both deletions may act together to further attenuate this virus in normal tissues, if necessary.

This new oncolytic vector was also shown to be capable of targeting and infecting human colorectal tumor explants ex vivo, and of destroying tumor cells by multiple mechanisms of action, one of which, to the best of our knowledge, has not been previously described. First, cancer-selective replication results in direct oncolysis. Second, as rechallenge of mice with tumors following complete responses to treatment resulted in tumor rejection, it appeared that the virus was capable of inducing an antitumor immune state within the animal. Induction of tumor-specific CTLs by oncolytic virus treatment was reported previously with HSV [31], but has not been shown for vaccinia virus. Although the exact mechanisms have not been proven, they are likely to include recruitment of antigen-presenting cells, induction of immunostimulatory cytokines, and release of tumor-associated and viral antigens following cell lysis, leading to in situ vaccination against the tumor. Finally, we report the infection of tumor-associated endothelial cells by the oncolytic virus, resulting in reduced tumor vascularity. Tumor-associated endothelial cell lysis can lead to tissue factor release and intratumoral vascular thrombosis. Endothelial cells are attractive targets for oncolytic viruses, given their accessibility to infection by intravascular virus [32]. Tumor-associated endothelial cells may be specifically susceptible to this vaccinia mutant for several reasons [33]. First, these cells tend to be hyperproliferative, and therefore may be generally more susceptible to vaccinia infection. Second, epithelial growth factor (EGF) receptors are frequently expressed on these cells. Vaccinia replication is enhanced by EGF receptor binding and activation by vaccinia growth factor (VGF). However, further research is needed to elucidate the mechanisms involved and to take full advantage of this novel antitumoral approach. It will also be interesting to determine whether tumor–endothelial cell targeting occurs with other vaccinia virus mutants and/or other oncolytic viruses.

Recombinant IFN-β has been administered systemically for the treatment of several cancer types. The protein has been delivered by intramuscular, intravenous, or intratumoral routes, with common toxicities including myelosuppression, transaminitis, and neurotoxicity (include seizures), indicating that localized, tumor-specific delivery of the cytokine would be desirable. Antitumoral efficacy was reported, however, both in patients with brain tumors (including glioblastoma multiforme) [34,35] and in a patient with colorectal carcinoma [36]. IFN-β therefore represents a promising cytokine for use in cancer therapy. However, because the effects of the recombinant protein are locally mediated and are short-lived in vivo, and its systemic administration leads to toxicity, expression of IFN-β from a gene therapy or oncolytic virus within the tumor represents a promising means to apply this cytokine [37]. However, previous approaches have suffered from a lack of targeted gene delivery [38,39].

The expression of IFN-β from *WR-delB18R* therefore represents a promising strategy. Transgene “arming” of oncolytic viruses has frequently been utilized to enhance antitumoral efficacy and for noninvasive imaging purposes [10]. However, in addition to increasing the antitumor effects of the virus, IFN-β expression serves to further reduce viral replication in normal tissues. To date, vaccinia virus–expressed transgenes have not been utilized to inhibit viral replication and enhance clearance from normal tissues; because IFN-β has potent antiviral properties, we predicted viral inhibition would occur in this case. A similar strategy with IFN-α was recently described for an oncolytic adenovirus vector [39]. However, this vector did not demonstrate systemic delivery or efficacy potential, and efficacy was limited even with multiple (more than ten) intratumoral injections. Vector replication and selectivity were not studied in normal nonimmortalized cells, and no primary human tissue was tested as reported in this study. Cancer selectivity was not studied in vivo, either, because tumor-free animals were studied for toxic effects to the liver only. In addition, because vaccinia genes expressed from early/late promoters will be expressed at low levels even during nonproductive infection of resistant cells, a small amount of IFN-β will be expressed in any normal tissues exposed to the virus, allowing the early production of an antiviral state. Of note, these levels are nontoxic, and high levels of gene expression are linked tightly to viral replication within tumor tissue. In this way, transgene expression will be linked to permissive infection, and thereby restricted to tumor cells.

The *IFN-β* gene was inserted into the *TK* gene, as this deletion has also been demonstrated to be tumor targeting [25]. The resulting virus, *JX-795*, has deletions in both *B18R* and *TK* genes, and expressed IFN-β, as well as luciferase, for preclinical imaging purposes. It was shown to be highly specific for cancer cells in vitro, without the requirement for addition of exogenous cytokine. *JX-795* was also found to produce high levels of IFN-β in vivo, which remained localized within the tumor. Viral gene expression was also highly tumor-restricted in vivo, and so this report represents the first description of a system for the systemic delivery of type-I IFN to tumors. Furthermore, this virus was capable of effectively destroying established tumors in mouse models. We therefore demonstrated that *JX-795* is highly tumor selective and capable of potent antitumor effects in vivo. The highly tumor-restricted *luciferase* gene expression seen with *JX-795* indicates that this vector could also be used as a gene-delivery vehicle for any further therapeutic transgenes whose expression might lead to toxicity if expressed in any nonmalignant tissues.

In addition to *B18R*, vaccinia virus expresses several other type-I IFN-resistance proteins. These include several intracellular proteins that prevent production of IFN from infected cells through inhibition of PKR (e.g., E3L and K3L), or blockade of nuclear factor-κB (NF-κB) activation and interferon-regulatory factor (IRF) signaling (A52R, A46R, and N1L). In addition, additional proteins are expressed that may prevent infected cells from responding to IFN by blocking STAT1 signaling (H1L) [20]. Future research may demonstrate that the normal tissue clearance of *JX-795* is further enhanced by deletion of one or more of these genes or regions within these genes. However, although clearance from normal tissues is advantageous, overly rapid clearance from tumor tissue may reduce efficacy. A balance will need to be achieved for future viruses derived from *JX-795*.

The rational design of oncolytic viruses combining tumor-targeting viral deletions with specific transgenes capable of complimenting, or even synergizing with, the phenotype of the attenuated virus represents a promising strategy for the design of virotherapeutics. In addition, the potential to destroy the tumor by a multitude of different mechanisms, as seen with oncolytic vaccinia strains, and the targeting of not only the malignant cells, but also other cells (e.g., endothelial and immune cells) within the tumor environment, may be the most effective approach to applying biological therapies.

## Abbreviations

CTL: cytotoxic T lymphocyte
GFP: green fluorescent protein
LPS: lipopolysaccharide
mIFN-β: murine interferon-beta
MOI: multiplicity of infection
MV: myxoma virus
NHBE cell: normal human bronchial epithelial cell
PFU: plaque-forming unit
SAEC: small airway epithelial cell
WR: Western Reserve
